# MUC-1 gene is associated with prostate cancer death: a 20-year follow-up of a population-based study in Sweden

**DOI:** 10.1038/sj.bjc.6603944

**Published:** 2007-08-28

**Authors:** O Andrén, K Fall, S-O Andersson, M A Rubin, T A Bismar, M Karlsson, J-E Johansson, L A Mucci

**Affiliations:** 1 Department of Urology, Örebro University Hospital, Örebro 701 85, Sweden; 2 Department of Medical Epidemiology and Biostatistics, Karolinska Institutet, Stockholm 171 77, Sweden; 3 Department of Pathology, Brigham and Womens Hospital, Boston, MA 02215, USA; 4 Department of Pathology, Örebro University Hospital, Örebro 701 85, Sweden; 5 Department of Medicine, Channing Laboratory, Harvard Medical School/Brigham and Women's Hospital, Boston, MA 02215, USA; 6 Department of Epidemiology, Harvard School of Public Health, Boston, MA 02215, USA

**Keywords:** prostate cancer, MUC-1, population-based, prognostic marker, twenty year follow-up, anti-adhesion

## Abstract

Anti-adhesion mucins have proven to play an important part in the biology of several types of cancer. Therefore, we test the hypothesis that altered expression of MUC-1 is associated with prostate cancer progression. We retrieved archival tumour tissue from a population-based cohort of 195 men with localised prostate cancer (T1a-b, Nx, M0) that has been followed for up to 20 years with watchful waiting. Semi-automated, quantitative immunohistochemistry was undertaken to evaluate MUC-1 expression. We modelled prostate cancer-specific death as a function of MUC-1 levels accounting for age, Gleason grade and tumour extent, and calculated age-adjusted and multivariate adjusted hazard ratios (HR). Men that had tumours with an MUC-intensity lower or higher than normal tissue had a higher risk of dying in prostate cancer, independent of tumour extent and Gleason score (HR 5.1 and 4.5, respectively). Adjustment for Gleason grade and tumour stage did not alter the results. Men with a Gleason score ⩾7 and MUC-1 deviating from the normal had a 17 (RR=17.1 95% confidence interval=2.3–128) times higher risk to die in prostate cancer compared with men with Gleason score <7 and normal MUC-1 intensity. In summary, our data show that MUC-1 is an independent prognostic marker for prostate cancer death.

Cancer metastasis involves the dysregulation of a complex interplay of multiple pathways. One critical step towards metastatic potential involves the detachment of tumour cells from the surrounding environment. Anti-adhesion molecules inhibit the cell–cell and cell–extra-cellular matrix interactions, and may promote development of metastatic disease by down regulating cellular adhesion.

The mucin family of anti-adhesion molecules have been implicated in the biological behaviour and progression of several types of cancer (Bresalier *et al*, 1991; Gendler and Spicer, 1995). The mucin *MUC-1*, which is expressed at the apical cell surface of many normal secretory epithelial cells (Ho *et al*, 1993), contains an extra-cellular domain that extends above most other cell membrane-associated proteins (Hilkens *et al*, 1992; Hilkens *et al*, 1995). As such, *MUC-1* has been suggested to prevent adhesion and to promote development of metastatic disease. In prostate cancer, overexpression of *MUC-1* in tissue has been correlated both with higher Gleason grade and advanced tumour stage (Kirschenbaum *et al*, 1999). One study has, furthermore, suggested that *MUC-1* expression may predict prostate cancer recurrence after prostatectomy (Lapointe *et al*, 2004), although these results have been challenged by others (O'Connor *et al*, 2005; Zellweger *et al*, 2005). The disparate findings may in part be explained by the use of PSA-recurrence as a measure of outcome, as biochemical failure does not necessarily herald prostate cancer death (Jhaveri *et al*, 1999).

In the present study, we test the hypothesis that altered tumour expression of *MUC-1* is associated with prostate cancer death. We nest the study in a population-based cohort of men with localised prostate cancer who have been followed prospectively for more than 20 years.

## MATERIALS AND METHODS

The study population is a cohort of all cases of early prostate cancer (T1a-b, Nx, M0) diagnosed at the Örebro University Hospital, Sweden between 1977 and 1991 by transurethral resection of the prostate or transvesical adenoma enucleation for symptomatic benign prostatic hyperplasia (Andren *et al*, 2006). As no private institutional care was available in the region at the time and as the population was required to seek care within their county of residence, the cohort can be considered population-based. Baseline evaluation at diagnosis included physical examination, chest radiography, bone scan and skeletal radiography (if needed). Nodal staging was not carried out. In accordance with standard practice at that time in Örebro, these patients were initially followed expectantly (‘watchful waiting’) (Johansson *et al*, 2004). Patients received clinical exams, laboratory tests and bone scans every 6 months during the first 2 years after diagnosis and subsequently at 12-month intervals. Patients that developed metastases, as judged by bone scan, were treated with androgen deprivation therapy if they exhibited symptoms.

Cause of death in the cohort was determined by review of medical records by the study investigators. An autopsy was performed if the cause of death was not clear. A validation study regarding cause of death compared with the Swedish Death Register showed greater than 90% concordance, with no systematic under- or over-reporting of any cause of death. Follow-up of the cohort with respect to mortality was 100%, and no patients were lost to follow up.

**Figure 1:**
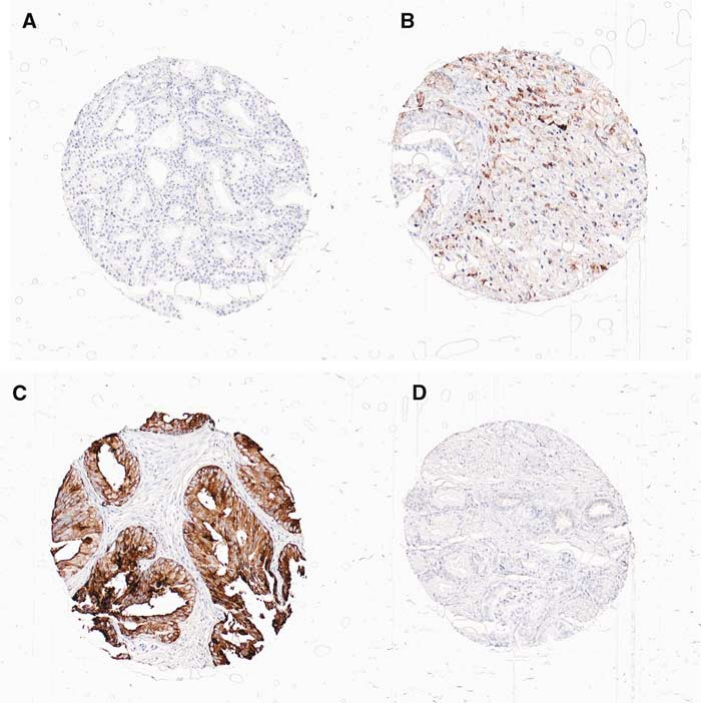
Tissue micro array analysis of MUC-1 immunohistochemistry: selected images of TMA cores representing normal, high and low MUC-1 intensity (**A**, **B**). Normal MUC-1 intensity (**C**, **D**). High (**C**) and Low (**D**) MUC-1 intensity.

We retrieved archival tissue specimens (formalin-fixed paraffin embedded) and H&E slides from all 240 cases in the cohort, and we had sufficient tumour tissue available for a total of 195 cases. Histological examination was performed by one pathologist (MAR) for Gleason grade who also assessed tumour extent by calculating the ratio of the number of chips with cancer and the total number of chips (Humphrey and Vollmer, 1988). High-density tissue micro arrays (TMAs) were assembled from the TUR-P specimens using the manual tissue arrayer (Beecher Instrument, Silver Spring, MD, USA) as described previously (Perrone *et al*, 2000). Two representative cores were taken from each tumour specimen. Benign tissue was also included on the TMA. After construction, a 4 *μ*m section was cut and stained with standard biotin–avidin complex immunohistochemistry antibodies (Mucin 1(VU4H5): sc 73–13) to evaluate *MUC-1*. Semi-automated, quantitative immunohistochemistry was undertaken using the Chromavision system, and protein intensity was measured on a scale from 0 to 255 (Figure 1A–D).

Through March 2003, with up to 23 years of follow-up (mean=9, median=8), 37 (19.0%) patients in this cohort had died of prostate cancer. The remaining patients were considered censored having either died of other causes (132 or 67.7%) or were still alive without disease at time of last follow-up (26 or 13.8%). We estimated person-time in the cohort as time between date of diagnosis to cancer death, or censored at death from other cause or end of follow-up (October 2003). We used Cox regression to model time to prostate cancer death as a function of *MUC-1* levels accounting for age, Gleason grade and tumour extent. For each individual, we calculated the mean intensity across the two cores. We defined normal MUC-1 intensity as the mean intensity in benign prostate tissue±0.25 standard deviations (s.d.). Individuals whose *MUC-1* tumour expression was within the normal range represented the reference group. Individuals whose tumour expression was above or below normal were so classified. Using Cox-regression, we calculated age-adjusted and multivariate adjusted hazard ratios (HR). We tested for linear trend for continuous variables using the McPhearson's test. Furthermore, we calculated the sensitivity and specificity of *MUC-1* protein expression as a predictor of prostate cancer death to explore its usefulness as a biomarker of prostate cancer outcomes.

## RESULTS

**Figure 2:**
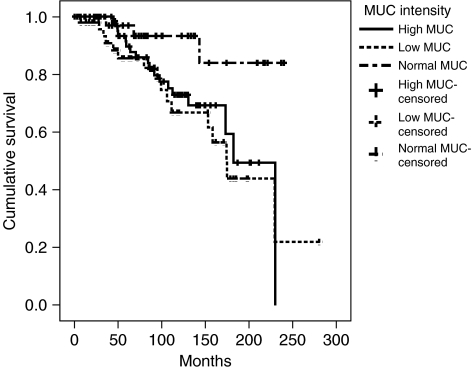
Survival curves of 195 patients according to high-, low- and normal MUC-1 intensity.

**Figure 3:**
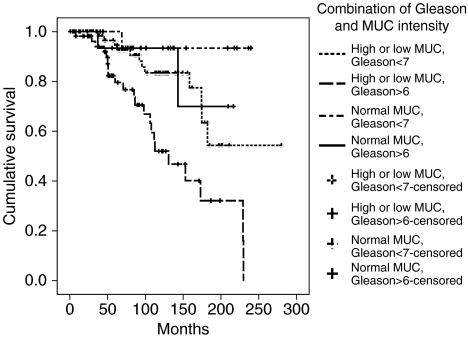
Survival curves of 195 patients according to combination of Gleason score and MUC-1 intensity.

The baseline characteristics of the 195 patients are presented in Table 1. Mean *MUC-1* expression for all 195 patients was 107.3 (range 95–179, s.d. 10.2). Of the 43 patients that had an *MUC-1* intensity close to normal tissue (Figures 1 and 2) (102.5–106), three (7%) died of prostate cancer, compared with 34 (23%) of the 152 patients that deviated from the normal *MUC-1* intensity (Figure 2 and 3). As illustrated in Table 2, there was no correlation between Gleason score and *MUC-1* intensity (*P*-value 0.8), whereas there was a tendency to correlation between tumour extent and MUC-1 (*P*-value 0.08).

The age-adjusted risk of dying of prostate cancer with respect to *MUC-1*, Gleason score and tumour extent is presented in Table 3. The risk of dying of prostate cancer was four times higher among those with a higher (3.9 (95% confidence interval (CI)=1.1–14)) or lower (3.8 (95%CI=1.1–13)) MUC-1 expression than among those with an *MUC-1* expression within the normal range. After adjusting for tumour extent and Gleason score, the effect of MUC-1 was even stronger (HR 5.1 (95%CI=1.4–18) and 4.5 (95%CI=1.3–15), respectively), indicating that *MUC-1* predicts prostate cancer death independently of clinical parameters.

We further cross-classified participants on *MUC-1* and Gleason score. The group with Gleason score ⩾7 and MUC-1 lower or higher than normal had a 17 (HR=17.1 (95%CI=2.3–128)) times higher risk of prostate cancer death compared with tumours with Gleason score <7 and normal *MUC-1* intensity (Table 4).

We further assessed the ability of MUC-1 expression (deviating from the normal range) to correctly classify prostate cancer cases as indolent or lethal (defined as progressing to metastases and/or death). The sensitivity for *MUC-1* as predictor of lethal prostate cancer was 0.91, whereas the specificity was 0.25 (Table 5). When combined with information on Gleason score, the specificity increased to 0.75 but the sensitivity decreased to 0.56.

## DISCUSSION

In this population-based cohort of men with localised prostate cancer cases (T1a-b, Nx, M0), we found that *MUC-1* expression at diagnosis was a predictor of prostate cancer death. Individuals with dysregulated (either over or under) *MUC-1* expression had a four- to five-fold increased risk of dying of prostate cancer, independent of clinical parameters. We further examined the accuracy of using *MUC-1* as a test for prostate cancer progression and found that using *MUC-1* alone resulted in a reasonable sensitivity but poor specificity. The addition of information on Gleason Score improved the specificity of the *MUC-1* biomarker, but at the cost of a lower sensitivity. Still, these data suggest that *MUC-1* may be promising to include in a panel of molecular markers to distinguish aggressive disease from indolent at diagnosis.

These data are in line with the accumulating evidence of the role of *MUC-1* in the cell–cell interaction. On the one hand, overexpression of *MUC-1* has been shown to increase the metastatic potential of the cancer cells (Evangelou *et al*, 2002; Schut *et al*, 2003; Huang *et al*, 2004). On the other, Kontani *et al* (2001) suggested that the loss of MUC-1 expression or modulation of its antigenicity might cause cancer cells to be unresponsive to the effect of cytotoxic T lymphocytes. These competing mechanisms provide possible explanations for our findings that both over- and underexpressions of *MUC-1* increases the risk of prostate cancer progression.

In this study, we could not confirm the findings of Kirschenbaum *et al* (1999) of a correlation between *MUC-1* expression and tumour differentiation. Differences in the assessment of *MUC-1* expression may explain the diverging results however; although we evaluated staining intensity, Kirchenbaum *et al* examined staining patterns (apical and diffuse). Our data furthermore confirm the results of Lapointe *et al* (2004) demonstrating that *MUC-1* is an independent prognostic marker that adds prognostic information over and above known risk factors of grade and stage.

The strengths of the study include the long-term and complete follow-up of more than 20 years. A unique feature is that the cohort includes a sizeable number of prostate cancer deaths, which allowed for us to evaluate an important clinical outcome. The fact that the cases had not received any curative treatment further enabled us to explore the effect of dysregulated *MUC-1* expression in the natural course of the disease. We employed high-density TMAs, which represent an efficient approach for immunohistochemical analysis, that also reduce potential batch-to-batch variation in staining. Moreover, the Chromavision system provided an automated assessment of protein intensity on a continuous scale. Our cases were diagnosed before introduction of PSA screening in the population, and thus PSA levels at diagnosis were not available. Although controlling PSA levels could attenuate the association between *MUC-1* and prostate cancer death, it is unlikely that it would fully explain the relationship.

In summary, these data show that *MUC-1* is an independent prognostic marker for prostate cancer death. Furthermore, although based on small numbers, our data suggest that *MUC-1* expression together with Gleason grade provide substantial information to distinguish prostate cancer outcomes.

As the accuracy of *MUC-1* alone or together with the Gleason Score in predicting prostate cancer progression was low, we conclude that its use as a single biomarker in clinical decision making is limited. Yet, we believe that alterations in MUC-1 expression may be useful as part of a composite set of biomarkers in accurately predicting prostate cancer outcome.

## Figures and Tables

**Table 1:** Characteristics of 195 patients with incidental prostate cancer (T1a-b, Nx, M0) who received no initial treatment, according to age, Gleason score, tumour extent and MUC-1 expression at time of diagnosis 1977–1991

	***N***	**Prostate cancer deaths**	**Deaths by other causes**	**Alive**	**Mean survival (month)**	**Minimum survival (month)**	**Maximum survival (month)**
*Age*							
<70	56	13	27	16	138	16	280
>70	139	24	105	10	90	1	272
							
*Gleason score*							
4	3	—	1	2	132	56	167
5	7	3	3	1	122	5	235
6	107	10	77	20	118	1	284
7	53	12	38	3	96	1	230
8	22	11	11	0	73	4	229
9	3	1	2	0	49	17	85
							
*Percentage of chips with cancer*							
<5%	80	6	58	16	102	1	237
6–25%	85	18	57	10	97	1	280
26–50%	13	6	7	0	85	17	153
>50%	17	7	10	0	54	4	143
							
*MUC-1*							
Normal	43	3	35	5	118	4	284
Low	68	16	38	14	108	1	280
High	84	18	59	7	100	1	238

**Table 2:** Correlation between MUC-1 intensity expression and age, Gleason score and tumour volume

		**MUC-1 intensity**		
**Factor**	**Normal *N* (%)**	**Low *N* (%)**	**High *N* (%)**	***P*-value**
Age <70	10 (23)	25 (37)	21 (25)	0.188
Age >70	33 (77)	43 (63)	63 (75)	
Gleason 4–6	25 (58)	41 (60)	51(61)	0.826
Gleason 7	13 (30)	20 (29)	20 (24)	
Gleason 8–9	5 (12)	7 (10)	13(16)	
Percent chips <5%	15 (35)	23 (34)	42 (50)	0.085
Percent chips 5–25%	23 (54)	33 (49)	29 (35)	
Percent chips 25–50%	0 (0)	5 (7)	8 (10)	
Percent chips >50%	5 (12)	7 (10)	5 (6)	

**Table 3:** HR and 95% CI for prostate cancer death in relation to protein expression of MUC-1 in tumour tissue from patients with localised prostate cancer

	***N* a**	**Prostate cancer deaths**	**Crude HR (95%CI)**	**HR^b^** **(95%CI)**
*MUC-1 (intensity)*				
Normal (102.5–106)	43	3	1.0	1.0
Low (<102.5)	68	16	3.9 (1.1–4)	5.1 (1.4–18)
High (>105.5)	84	18	3.8 (1.1–13)	4.5 (1.3–15)

CI=confidence interval; HR=hazard ratio.

a A total of 195 patients were assayed for MUC-1.

b Adjusted for age, Gleason score and tumour extent.

**Table 4:** Hazard ratio (95% CI) of prostate cancer death associated with MUC-intensity and Gleason score, cross classified

	**Normal MUC-intensity**	**Aberrant MUC-intensity**
	**PC death/total *N* HR (95%CI)**	**PC death/total *N* HR (95%CI)**
Gleason <7	1/25 Ref.	12/92 3.8 (0.5–29)
Gleason ⩾7	2/18 3.8(0.3–43)	22/60 17.1 (2.3–28)

**Table 5:** Sensitivity, specificity, PPV and NPV of Gleason grade and MUC-1 intensity in predicting prostate cancer death

**Gleason score and MUC-1 intensity**	**Sensitivity**	**Specificity**	**PPV**	**NPV**
Aberrant MUC-1 intensity	0.91	0.25	0.22	0.93
Gleason >6 and aberrant MUC-1 intensity	0.59	0.76	0.37	0.89

NPV=negative predictive value; PPV=positive predictive value.
